# Noninvasive Ventilation-assisted Bronchoscopy in High-risk Hypoxemic Patients

**DOI:** 10.5005/jp-journals-10071-23219

**Published:** 2019-08

**Authors:** Mrinal Sircar, Onkar K Jha, Gurmeet S Chabbra, Sandip Bhattacharya

**Affiliations:** 1,2 Department of Pulmonology and Critical Care, Fortis Hospital, Noida, Uttar Pradesh, India; 3 Department of Respiratory and Sleep Medicine, QRG Central Hospital and Research Centre, Faridabad, Haryana, India; 4 Department of Critical Care, Asian Institute of Medical Sciences, Faridabad, Haryana, India

**Keywords:** Bronchoscopy, FOB, NIV, Noninvasive ventilation, Respiratory failure

## Abstract

**Background and aims:**

Hypoxemic patients undergoing fiber-optic bronchoscopy (FOB) are at risk of worsening of respiratory failure requiring mechanical ventilation due to FOB procedure itself and its complications. As patients with respiratory failure are frequently managed by non-invasive ventilation (NIV); feasibility of FOB through NIV mask has been evaluated in some studies to avoid intubation. We describe here our own case series.

**Materials and methods:**

Clinical data of 28 FOB done through NIV mask in 27 intensive care unit (ICU) patients over 6 years period at our center was collected retrospectively and analysed.

**Results:**

Study comprises 27 (17 male; 52±21.6 years age) hypoxemic (PaO_2_ 71.3±14.2, on NIV and oxygen supplementation) patients. All FOB were done at bedside, 15 of them were given sedation for the procedure. Twenty four patients had bronchoalveolar lavage (BAL); three underwent bronchial biopsies, four brush cytology and seven transbronchial biopsies. In 10 patients lung or lobar collapse was reversed. There was no significant change between pre and post bronchoscopy ABG parameters except for improved post FOB PaO_2_ (*p* = 0.0032) and SpO_2_ (*p* = 0.0046). One patient (3.57%) developed late pneumothorax and 3 patients (10.7%) had bleeding after biopsy. Prior to bronchoscopy 17 (16 BIPAP, 1 CPAP) patients were already on NIV. Two patients required mechanical ventilation 6 hours after FOB due to subsequent clinical deterioration but could be weaned off later. One patient died on third day after FOB from acute myocardial infarction.

**Conclusion:**

Hypoxemic patients in ICU can safely undergo bedside diagnostic and simple therapeutic bronchoscopy with NIV support while mostly avoiding intubation and with low complication rates.

**How to cite this article:**

Sircar M, Jha OK, Chabbra GS, Bhattacharya S. Noninvasive Ventilation-assisted Bronchoscopy in High-risk Hypoxemic Patients. Indian J Crit Care Med 2019;23(8):363-367.

## INTRODUCTION

Patients with respiratory failure in intensive care unit (ICU) may require fiber-optic bronchoscopy (FOB). However, FOB itself can lead to hypoxemia, hypotension, tracheobronchial bleeding or cardiac arrhythmias.^1,2^ Although empirical treatment is possible, etiological diagnosis is necessary for targeted treatment.^3^ Strategies for microbiological diagnosis may include sputum or endotracheal aspirate examination. In non-intubated patients FOB may be done via laryngeal mask airway^4^ or by intubating them.^5^ These invasive methods may be avoided by using non-invasive ventilation (NIV) during FOB.^5–18^ An alternative technique using high-flow nasal cannula oxygen therapy during FOB is also emerging.^19^

Bronchoscopy with NIV support is relatively less used technique with small number of published papers and only one from India.^16^ We have been using this technique and our experience over a period of six years is presented here.^20^

## MATERIALS AND METHODS

The study population comprised of 27 patients undergoing 28 bedside FOB procedures on NIV during a period of six years in a medical–surgical ICU. Intent of bronchoscopy was therapeutic in 10 patients with atelectasis (four patients with one lung and six with lobar collapse) that were unresponsive to physiotherapy and was diagnostic in the remaining eighteen.

Of these 28 patients’ episodes, 17 were already on NIV (16 on BIPAP and one on CPAP). All these patients were observed to desaturate even with a brief discontinuation (<10 minutes) of NIV and were therefore considered unfit for bronchoscopy off NIV. The patient on CPAP was electively switched to BIPAP for FOB. The remaining 11 patients (included four with lung collapse and rest with diffuse lung diseases) had PaO_2_/FiO_2_ ratio <300, respiratory rate >30 per min and using accessory muscles of respiration but were not candidates for immediate intubation. These 11 patients were put electively on BIPAP, based on clinical judgement, one hour before FOB.

**Fig. 1:**
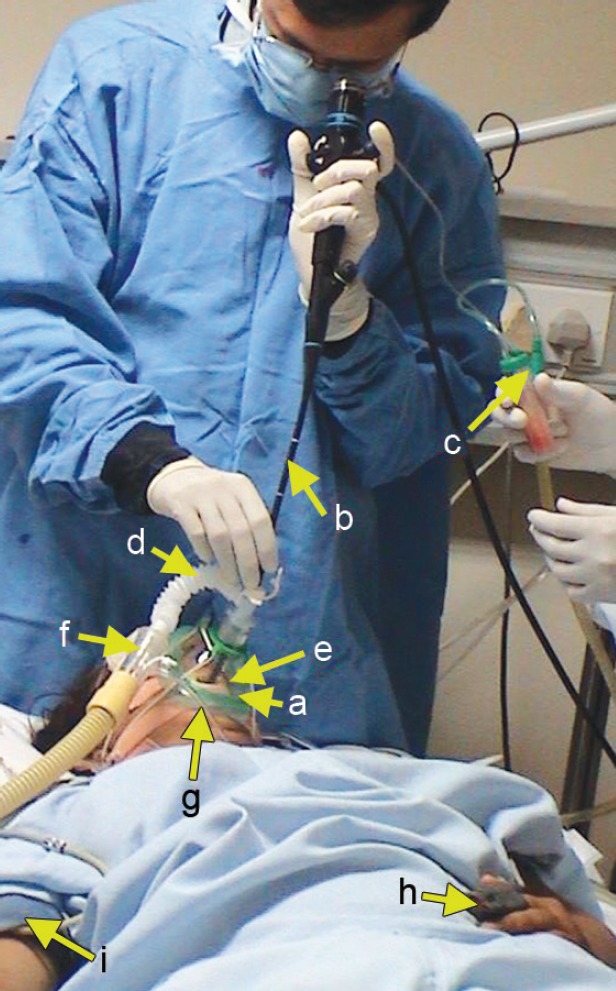
Fiber-optic bronchoscopy through non-invasive ventilation. (a) Anesthesia face mask; (b) Fiber-optic bronchoscope; (c) Trap for BAL collection; (d) Catheter mount; (e) Bite guard; (f) Oxygen enrichment connector; (g) Oxygen tubing; (h) Pulse oximeter; (i) Noninvasive BP cuff

Noninvasive ventilation was delivered through a tight fitting anaesthesia face mask (Intersurgical Ltd., Berkshire, UK), kept in place using adjustable straps. This mask was then attached to a catheter mount with inbuilt bronchoscopy port, an Oxygen enrichment connector with inbuilt expiratory port (Respironics; Murrysville, PA) and a single limb NIV circuit that was attached to BiPAP-S/T30 machine (BiPAP; Respironics; Murrysville, PA) (Fig. 1). Oxygen was bled into the NIV circuit and flow was adjusted to keep SpO_2_ above 90% during the bronchoscopy procedure.

Sixteen patients were already on BIPAP and they continued on their preexisting setting of IPAP of 12–20 cm H_2_O and EPAP of 4–8 cm H_2_O. For the remaining 12 (including patient on CPAP) patients’ initial settings were IPAP of 12 cm H_2_O and EPAP of four cm H_2_O and this was adjusted based on clinical response. All patients were also put on a backup rate of 12 breaths per min. Arterial blood gas(ABG) analysis was obtained after one hour when stable clinical parameters were achieved for these 12 patients, as also for all other patients, before proceeding to FOB. ABG was repeated one hour after FOB procedure.

All patients received topical anesthesia (10% lignocaine throat spray and 2% lignocaine via bronchoscope). Sedation was used as infusion during 15 bronchoscopies (1% Propofol in 14 patients and Midazolam in one patient).^15^ All patients had bedside ECG, (non-invasive or arterial) blood pressure and pulse oximetry monitoring during the procedure. A critical care physician provided the sedation and monitored the patient during and after FOB procedure. All bronchoscopies were done by consultant pulmonologists. Bronchoscopy was done through nasal passage in 23 patients and through oral route in five patients (using a bite guard inside the NIV mask). Fiber-optic bronchoscope used (Olympus BF-1T30) had 6.0-mm outer diameter with 2.8 mm instrument channel. Chest X-ray was done in all patients two hours after FOB procedure.

## RESULTS

Twenty-eight FOB procedures (in 27 patients, 18 males, mean +SD age 52+21.6 years) were done using NIV support. Demographic details are shown in (Table 1). Indications and results of bronchoscopy are given in (Table 2).

Collapsed lung or lobes were successfully opened up in all 10 patients by repeated saline and N-acetyl cysteine instillations and suctioning. Broncheoalveolar lavage (BAL) samples were obtained in 24 patients. Bronchial biopsies were done in three patients, brush cytology in four patients and transbronchial biopsies (TBLB) (without using C-arm) in seven patients.

**Table 1:** Characteristics of study population

*Parameters*	*Value*
Age (years) mean ± SD	52 ± 21.6
Gender n (%): Male Female	18 (64.3) 10 (35.7)
Comorbid Illnesses *n*(%): Coronary artery disease Cerebral vascular accident Hypertension Chronic renal failure Bronchial arthma Diabetes mellitus Obstructive sleep apnea Interstitial lung disease	1 (3.57) 3 (10.7) 4 (14.3) 1 (3.57) 1 (3.57) 1 (3.57) 1 (3.57) 3 (10.7)
Immunosupression n(%): Malignancy hematological Malignancy solid organ Long-term steroid	1 (3.57) 3 (10.7) 4 (14.3)
Reason of respiratory failure *n*(%): Pneumonia ARDS Atelectasis Pulmonary hemorrhage Diffuse infiltrates	6 (21.4) 1 (3.57) 14 (50) 2 (7.14) 5 (15.3)
Type of bronchoscopy *n*(%): Diagnostic Therapeutic	18 (64.2) 10 (35.7)
Use of NIV *n* (%): Preprocedure Intraprocedure Postprocedure	16 (57.1) 28 (100) 19 (67.9)
Route of bronchoscopy *n* (%): Nasal Oral	23 (82.1) 5 (21.7)
Postprocedure complication *n*(%): Pneumothorax Bleeding Mechanical ventilation	1 (3.57) 4 (14.3) 2 (7.14)

All procedures were well tolerated; no patient needed endotracheal intubation and no hemodynamic instability occurred during FOB. Sedation was well tolerated by patients. Three patients had significant bleeding during FOB that was controlled with bronchoscopic instillation of adrenaline and cold saline. Routine postprocedure chest X-rays after two hours did not reveal any pneumothorax. There was significant improvement in oxygenation noted in post bronchoscopy ABGs in comparison with pre-FOB ABG (Table 3).

All patients on pre-FOB NIV (17 patients) remained on BIPAP afterwards too without requiring intubation. Of the remaining 11 patients, NIV could be discontinued 1–2 hours after FOB in eight patients; one of them developed acute respiratory distress six hours after FOB including TBLB. A repeat chest X-ray revealed a delayed pneumothorax requiring emergent intercostal tube drainage and invasive mechanical ventilation. The patient could be successfully extubated after two days. However, a day later he died of myocardial infarction. This patient had history of myocardial infarction five year back.

**Table 2:** Indications and results of fiber-optic bronchoscopy

*Case #*	*FOB Indication*	*Sedation*	*Bronchoscopy result*	*Microorganism*	*Complication*
1	Atelectasis	No sedation	Infection	*Pseudomonas aeruginosa*	None
2	Nodular infiltrates	Propofol	Infection, neoplasia (carcinoid)	*M. Tuberculosis, Candida albicans*	Late pneumothorax, Mechanical ventilation
3	Bilateral infiltrates	Propofol	Infection	*E. coli*	None
4	Atelectasis, blunt trauma abdomen (post-laparotomy)	No sedation	Atelectasis	Stains and culture negative	None
5	Atelectasis	No sedation	Infection	*Proteus mirabilis*	None
6	Atelectasis	Propofol	Infection	*Klebsiella pneumoniae*	None
7	Atelectasis, CRF with uremic pleural effusion	Propofol	Atelectasis	Stains and culture negative	None
8	Pneumonia	No sedation	Infection, atypical cell	Gram-negative bacilli	Bleeding
9	Atelectasis	No sedation	Infection, mucous plugging	*Klebsiella pneumoniae*	None
10	Atelectasis	No sedation	Infection	Gram-negative bacilli	None
11	Bilateral infiltrates	Propofol	Infection	*Pseudomonas, GPC, pseudohyphae*	Bleeding
12	Infiltrates, pulmonary nodule	No sedation	Infection, nonsmall cell carcinoma	*Candida albicans*	None
13	Atelectasis	No sedation	Infection,	*Candida albicans*	None
14	Atelectasis	Propofol	Infection	*E. coli, Klebsiella pneumoniae*	None
15	Atelectasis	Propofol	Infection, mucous plugging	Gram-negative bacilli	None
16	Pulmonary hemorrhage	Propofol	Chronic inflammation	Stains and culture negative	Mechanical ventilation
17	Atelectasis	No sedation	Infection	*Klebsiella pneumoniae*	None
18	Atelectasis	Propofol	Blood clot	Stains and culture negative	None
19	Pulmonary hemorrhage	Propofol	Interstitial lung disease (UIP)	Stains and culture negative	None
20	Organizing pneumonia	Propofol	Interstitial lung disease (BOOP)	Stains and culture negative	None
21	Pneumonia	Propofol	Copious secretions in tracheobronchial tree	Stains and culture negative	None
22	ARDS, pneumonia	No sedation	Infection	*Pseudomonas aeruginosa*	None
23	Atelectasis	No sedation	Infection	*E.coli*	None
24	Pneumonia	No sedation	Infection	*Acenetobacter baumannii*	Bleeding
25	Atelectasis	No sedation	Infection	*Candida albicans*	None
26	Stridor	Midazolam	Vocal cord edema	Stains and culture negative	None
27	Organizing pneumonia	propofol	Organizing pneumonia	Stains and culture negative	None
28	Bilteral infiltrates	propofol	Chronic inflammatory cells	Stains and culture negative	None

**Table 3:** Arterial blood gas parameters pre- and postfiber-optic bronchoscopy

*Parameters*	*Preprocedure*	*Postprocedure*	*Significance*
pH	7.404 ± 0.0634	7.404 ± 0.0556	0.3106
PaCO_2_ (mm Hg)	40.057 ± 8.528	40.021 ± 7.198	0.6186
PaO_2_ (mm Hg)	71.296 ± 14.160	80.079 ± 19.400	0.0032
HCO_3_ (mmol/L)	25.396 ± 5.096	25.064 ± 4.843	0.5599
SpO_2_ (%)	92.071 ± 3.761	93.607 ± 3.881	0.0046

Final bronchoscopic diagnosis for this patient was pulmonary tuberculosis and atypical carcinoid (Table 2, Case 2). Another patient with diffuse lung disease (Table 2, Case 16), who was put on NIV only for FOB but NIV could not be discontinued afterwards, needed elective mechanical ventilation six hours later due to worsening respiratory distress. He too could be extubated later.

## DISCUSSION

Fiber-optic bronchoscopy is extensively used in ICUs for diagnosis of pulmonary pathology and mucous plugging. However, bronchoscopy is often associated with temporary changes in gas exchange and lung mechanics.^21–25^

Fiber-optic bronchoscope occupies about 10% of tracheal cross section in nonintubated patients and this can increase work of breathing.^26^ In contrast, a 5.7 mm bronchoscope occupies 40% of a 9.0 mm internal diameter (ID) endotracheal tube and 66% of a 7.0-mm ID endotracheal tube^27^ potentially increasing the work of breathing even more.

Significant changes in gas exchange parameters occur during FOB, primarily during suctioning. Hypoxemia during or immediately after taking BAL is most common complication.^28^ PaCO_2_ increases by about 30% and PaO_2_ is decreased by 40%.^21^ Around 200–300 mL of tidal volume is lost during each suctioning period. These changes in blood gases result from decrease in lung volumes and loss of functional gas exchange surface during BAL and reflex bronchospasm due to stimulation of vagal receptors in upper airways. Desaturation during bronchoscopy has also been attributed to upper airway collapse that can be reversed by placement of nasopharengeal airway.^29^ The delay before normalization of gas exchange varies from about 15 minutes for normal lungs to several hours in those with parenchymal disease.^26,30^

Significant cardiopulmonary risk has been reported in up to 13% ventilated patients during bronchoscopy.^30^ The American Thoracic Society recommends avoiding BAL in patients spontaneously breathing with hypercapnia and/or hypoxemia and who cannot be corrected to at least PaO_2_ of 75 mm Hg or to SpO_2_ more than 90% with supplemental oxygen.^1^

Noninvasive ventilation has been successfully used for respiratory failure – it decreases work of breathing, improves lung mechanics, recruits closed alveoli, improves blood gases and avoids intubation and mechanical ventilation.^7,31^ As an extension, it has been used to perform FOB in patients with respiratory failure^14^ and it has been to shown to help in improving oxygen saturation and arterial oxygen tension and avoid invasive ventilation. Mechanisms for these are likely to be same as for treatment of respiratory failure with NIV. Further as a corollary with obstructive sleep apnea, NIV is likely to counter upper airway collapse during bronchoscopy.^32,33^ Moreover, as the presence of the bronchoscope within the trachea reduces its caliber and increases airway resistance and work of breathing, application of NPPV may compensate for this extra-work load, thereby improving the tolerability and safety of the bronchoscopic procedure. Small number of studies, mostly observational, have shown successful use of NIV assisted FOB in COPD,^6,18^ immunocompromized patients with pneumonia,^5^ acute respiratory failure with or without pneumonia in ICU,^13,15,16,34^ ARDS patients^17,35^ and non ICU hypoxemic patients.^12^ Few randomized control trials of NIV versus Oxygen supplementation^7,8^ and NIV versus mechanical ventilation^13^ have all reported benefit of from using NIV during FOB. Even upper gastrointestinal endoscopy,^36^ endoscopic retrograde cholangiopancreatography^37^ and transesophageal echocardiography^38^ have been successfully done under NIV support. In yet another study COPD patient in exacerbations with mild encephalopathy and inability to clear secretions were randomized to only NIV versus NIV combined with early FOB for bronchial toileting and latter had superior clinical outcomes.^18^

In our study too all 27 patients could successfully undergo 28 FOB procedure with NIV support without need for immediate intubation. This is one of the largest study evaluating feasibility of FOB in patients on NIV support. All published studies have reported NIV supported FOB guided BAL procedures. Only few studies like Maitre et al. (6 patients) and Chiner et al (eight patients) have reported bronchial biopsies while Agarwal R has reported transbronchial biopsies (six patients) during FOB on NIV support.^7,16,34^ Protected brushing has been reported by Chiner et al.^34^ In our study too we have reported obtaining BAL (24 patients), bronchial (three patients) and transbronchial (seven patients) biopsies as well as brushings (4 patients) safely.^20^ Three patients had significant bleed that was managed successfully while only one patient had (late) pneumothorax.

Two of our patients needed intubation and mechanical ventilation within 24 hours of bronchoscopy as have been reported earlier by other authors.^7,8,14,15,17,18^ The first one needed ventilation after developing a delayed pneumothorax that could be a possible complication of transbronchial biopsy procedure during preceding FOB or simply due to the underlying disease (pulmonary tuberculosis with atypical carcinoid) itself. The patient could be successfully extubated two days later after complete resolution of pneumothorax and resultant respiratory deterioration. The second patient needing mechanical ventilation after FOB had bilateral lung contusion after a road traffic accident and had only been subjected to BAL. Worsening of gas exchange is well known after BAL,^21,28^ which may or may not be prevented or reversed by application of NIV. This patient too could be extubated later.

Overall there was improvement in oxygenation although FOB can adversely affect it, perhaps due to inclusion of 10 patients who had resolution of their lung/lobe collapse.

All our patients were admitted in ICU. Bronchoscopy was therapeutic for 10 patients as they succeeded in opening up atelectatic areas. In 18 remaining instances FOB was primarily diagnostic in intent. Except for a few studies^11,12^ others too have done FOB with NIV support only for ICU patients.

We used sedation for 15 of 28 FOB procedures and it was well tolerated as has been reported by others in hypoxemic patients in general^39^ and during NIV supported FOB.^15,40^

Different interfaces have been used for FOB on NIV support including oro-nasal mask,^5,8,16,17^ full face mask,^14,18^ nasal mask with bite block covered by glove finger through which FOB is introduced,^10,34^ helmet mask,^9^ modified total face mask^12^ and Boussignac CPAP coupled to a face mask.^7^ No study till date has compared different types of masks.

We used a dedicated NIV device (BiPAP-ST) for our FOB procedures. Other studies have reported using either ICU ventilators to deliver NIV^5,8,13,14,16,17^ or dedicated NIV devices^17,34^ or even Boussignac CPAP device.^7^ All approaches seem to work equally well though no study has tried to compare different NIV devices.

Our study has shown that NIV can facilitate both diagnostic and therapeutic bronchoscopic procedures in selected hypoxemic ICU patients while mostly avoiding intubation. Not only BAL but biopsies and brushing could be done safely. Close monitoring of vital signs is required both before initiation of FOB and for extended periods afterwards as late complications can occur. All bronchoscopies were however performed by experienced pulmonologists while being monitored by intensivists with combined expertise to manage complications of FOB.
